# Supplementary material for: Morphological and chemotaxonomical characterization of some species of the genus Euphorbia L. in Jazan region, KSA
Source: PLoS One. 2025 Nov 5;20(11):e0335519. doi: 10.1371/journal.pone.0335519 (PMC12588485; doi:10.1371/journal.pone.0335519)
Supplement: Attachment — Submitted filename: PONE-D-25-25839 (1).pdf [file pone.0335519.s001.pdf]

PLOS ONE

Morphological and chemotaxonomical characterization of some species of the genus Euphorbia L. in Jazan region, KSA

--Manuscript Draft--

Manuscript Number:	PONE-D-25-25839
Article Type:	Research Article
Full Title:	Morphological and chemotaxonomical characterization of some species of the genus Euphorbia L. in Jazan region, KSA
Short Title:	Euphorbia L. with modern plant taxonomy
Corresponding Author:	Ahmed El-Shabasy, Ph.D.<br>Jazan University College of Science<br>Jazan, SAUDI ARABIA
Keywords:	: Adaptation; Alkaloids; Flavonoids; Habitat; HPLC; Phenolics; SPSS
Abstract:	This study assesses the classification of four Euphorbia species: E. ammak, E. fractiflexa, E. granulata, and E. hirta collected from different habitats to determine the interrelationships among them using traditional (morphological analysis) and modern (chemotaxonomical analysis) methods. The morphological characters included all aspects of plant life that represented as qualitative and quantitative parameters. Phytochemical analysis included the estimation of total phenolics, alkaloids, flavonoids, saponins, and tannins. HPLC was used to determine the phenolic pattern and confirm the chemotaxonomic study. HPLC analysis explained the presence of eleven phenolic and seven flavonoid compounds in the methanol extract of the four taxa of Euphorbia. All obtained data from the studied Operational Taxonomic Units (OTUs) was scored to be a binary matrix to establish similarity matrix and phenogram cluster. Duncan's range test was used to determine the significant of interrelations. The results revealed that all studied plant species were rich in phenolic constituents with different ranges. E. granulata was the most transition species among them. The study recommended in taking the adaptation and type of habitat into consideration in chemotaxonomy of plant species.
Order of Authors:	Yehia Hazzazi
	Mari Sumayli
	Ahmed El-Shabasy, Ph.D.
	Abeer Al-Andal
	Uzma Hanif
	Asmaa Khamis
	Emad Abada
	Sameh R. Elgogary
	Taha A. I. El-Bassossy
	Ahmed A. M. Abdelgawad
Additional Information:	
Question	Response
**Financial Disclosure**<br><br>Enter a financial disclosure statement that describes the sources of funding for the work included in this submission. Review the submission guidelines for detailed requirements. View published research	The author(s) received no specific funding for this work.

Powered by Editorial Manager® and ProduXion Manager® from Aries Systems Corporation

articles from *PLOS ONE* for specific examples.<br><br>This statement is required for submission and **will appear in the published article** if the submission is accepted. Please make sure it is accurate.<br><br>**Funded studies**<br>Enter a statement with the following details:<br>• Initials of the authors who received each award<br>• Grant numbers awarded to each author<br>• The full name of each funder<br>• URL of each funder website<br>• Did the sponsors or funders play any role in the study design, data collection and analysis, decision to publish, or preparation of the manuscript?<br><br>Did you receive funding for this work?	
**Competing Interests**<br><br>Use the instructions below to enter a competing interest statement for this submission. On behalf of all authors, disclose any competing interests that could be perceived to bias this work—acknowledging all financial support and any other relevant financial or non-financial competing interests.<br><br>This statement is required for submission and **will appear in the published article** if the submission is accepted. Please make sure it is accurate and that any funding sources listed in your Funding Information later in the submission form are also declared in your Financial Disclosure statement.<br><br>View published research articles from *PLOS ONE* for specific examples.	The authors have declared that no competing interests exist

**NO authors have competing interests**<br><br>Enter: *The authors have declared that no competing interests exist.*<br><br>**Authors with competing interests**<br><br>Enter competing interest details beginning with this statement:<br><br>*I have read the journal's policy and the authors of this manuscript have the following competing interests: [insert competing interests here]*<br><br>* typeset	
**Ethics Statement**<br><br>Enter an ethics statement for this submission. This statement is required if the study involved:<br><br>• Human participants<br>• Human specimens or tissue<br>• Vertebrate animals or cephalopods<br>• Vertebrate embryos or tissues<br>• Field research<br><br>Write "N/A" if the submission does not require an ethics statement.<br><br>General guidance is provided below. Consult the submission guidelines for detailed instructions. **Make sure that all information entered here is included in the Methods section of the manuscript.**	N/A

Powered by Editorial Manager® and ProduXion Manager® from Aries Systems Corporation

**Format for specific study types**<br><br>**Human Subject Research (involving human participants and/or tissue)**<br>• Give the name of the institutional review board or ethics committee that approved the study<br>• Include the approval number and/or a statement indicating approval of this research<br>• Indicate the form of consent obtained (written/oral) or the reason that consent was not obtained (e.g. the data were analyzed anonymously)<br><br>**Animal Research (involving vertebrate animals, embryos or tissues)**<br>• Provide the name of the Institutional Animal Care and Use Committee (IACUC) or other relevant ethics board that reviewed the study protocol, and indicate whether they approved this research or granted a formal waiver of ethical approval<br>• Include an approval number if one was obtained<br>• If the study involved *non-human primates*, add *additional details* about animal welfare and steps taken to ameliorate suffering<br>• If anesthesia, euthanasia, or any kind of animal sacrifice is part of the study, include briefly which substances and/or methods were applied<br><br>**Field Research**<br><br>Include the following details if this study involves the collection of plant, animal, or other materials from a natural setting:<br>• Field permit number<br>• Name of the institution or relevant body that granted permission	
**Data Availability**<br><br>Authors are required to make all data underlying the findings described fully available, without restriction, and from the time of publication. PLOS allows rare exceptions to address legal and ethical concerns. See the PLOS Data Policy and FAQ for detailed information.	Yes - all data are fully available without restriction

A Data Availability Statement describing where the data can be found is required at submission. Your answers to this question constitute the Data Availability Statement and **will be published in the article**, if accepted.<br><br>**Important:** Stating 'data available on request from the author' is not sufficient. If your data are only available upon request, select 'No' for the first question and explain your exceptional situation in the text box.<br><br>Do the authors confirm that all data underlying the findings described in their manuscript are fully available without restriction?	
**Describe where the data may be found in full sentences. If you are copying our sample text, replace any instances of XXX with the appropriate details.**<br><br>• If the data are **held or will be held in a public repository**, include URLs, accession numbers or DOIs. If this information will only be available after acceptance, indicate this by ticking the box below. For example: *All XXX files are available from the XXX database (accession number(s) XXX, XXX.)*.<br>• If the data are all contained **within the manuscript and/or Supporting Information files**, enter the following: *All relevant data are within the manuscript and its Supporting Information files.*<br>• If neither of these applies but you are able to provide **details of access elsewhere**, with or without limitations, please do so. For example:<br><br>*Data cannot be shared publicly because of [XXX]. Data are available from the XXX Institutional Data Access / Ethics Committee (contact via XXX) for researchers who meet the criteria for access to confidential data.*<br><br>*The data underlying the results presented in the study are available from (include the name of the third party*	All datasets were achieved, used, and analyzed in the current study. They are included in this manuscript.

and contact information or URL).<br>• This text is appropriate if the data are owned by a third party and authors do not have permission to share the data.<br><br>* typeset	
Additional data availability information:	

Powered by Editorial Manager® and ProduXion Manager® from Aries Systems Corporation

Manuscript Click here to access/download;Manuscript;Euphorbia.docx

Morphological and chemotaxonomical characterization of some species of the genus *Euphorbia* L. in Jazan region, KSA

Yehia Hazzazi[1], Mari Sumayli[1] , A. El-Shabasy[1*], Abeer Al-Andal[2], Uzma Hanif[3, 4], Asmaa Khamis[5], Emad Abada[1, 6] , Sameh R. Elgogary[7], Taha A.I. El-Bassossy[8], Ahmed A. M. Abdelgawad[7, 8]

1. Department of Biology, College of Science, Jazan University, P.O. Box. 114, Jazan 45142, Saudi Arabia

2. Department of Biology, College of Science, King Khalid University, Abha 61413, Saudi Arabia

3. Department of Botany, Government College University, Lahore Pakistan

4.Center for Functional Ecology: Science for People & Planet, Marine Resources, Conservation and Technology-Marine Algae Lab, Department of Life Sciences, University of Coimbra, 3000-456 Coimbra, Portugal

5. Department of Botany, Faculty of Science, Fayoum University, Fayoum, Egypt.

6. Environment and Nature Research Centre, Jazan University, P.O. Box 114, Jazan 45142, Saudi Arabia

7. Department of Physical sciences, Chemistry division, College of Science, Jazan University, P.O. Box. 114, Jazan 45142, Saudi Arabia.

8. Medicinal and Aromatic Plants Department, Desert Research Center, 11753, Cairo, Egypt.

* Corresponding author

Abstract. This study assesses the classification of four Euphorbia species: *E. ammak, E. fractiflexa*, *E. granulata*, and *E. hirta* collected from different habitats to determine the interrelationships among them using traditional (morphological analysis) and modern (chemotaxonomical analysis) methods. The morphological characters included all aspects of plant life that represented as qualitative and quantitative parameters. Phytochemical analysis included the estimation of total phenolics, alkaloids, flavonoids, saponins, and tannins. HPLC was used to determine the phenolic pattern and confirm the chemotaxonomic study. HPLC analysis explained the presence of eleven phenolic and seven flavonoid compounds in the methanol extract of the four taxa of *Euphorbia*. All obtained data from the studied Operational Taxonomic Units (OTUs) was scored to be a binary matrix to establish similarity matrix and phenogram cluster. Duncan's range test was used to determine the significant of interrelations. The results revealed that all studied plant species were rich in phenolic constituents with different ranges. *E. granulata* was the most transition species among them. The study recommended in taking the adaptation and type of habitat into consideration in chemotaxonomy of plant species.

Keywords: Adaptation, Alkaloids, Flavonoids, Habitat, HPLC, Phenolics, SPSS

1. Introduction

Phytochemicals are valuable guides for the most recent sciences like pharmacology, applied biotechnology and chemotaxonomy. The use of secondary metabolites from biosynthetic pathways as tannins, flavonoids, phenolics, saponins, and alkaloids is considered the proper way for determination of relations among plant species **[1]**. It can be used for solving the taxonomic problems and detecting the main gaps among transition interrelated plant species. Moreover, it can predict the medicinal beneficial of some other unstudied taxa with referring to their position in chematoxonomy. Phytochemical profiling of plant species for specific genus is feasible for different researchers to obtain rationale studies based on their different specializations **[2, 3]**.

Several studies reported the pharmacological activities **[4]** including antiproliferative **[5]**, cytotoxic **[6]**, antimicrobial **[7]**, antipyretic-analgesic **[8]**, inhibition of HIV1viral infection **[9]**, ethnomedicinal usage **[10, 11]**, and the structural diversity of isolated phytoconstituents **[12, 13]** of *Euphorbia* species.

Euphorbiaceae Juss. (Spurge family) is one of the largest families of flowering plants conspicuously throughout the tropics and composed of over 300 genera and 8000 species **[14]**.

Euphorbia L. is the largest genus in the family Euphorbiaceae with about 2000 species ranging from herbs to trees **[15]**. *Euphorbia* genus consists of species of great economic importance, which makes it a complex genus with great research potential.

There are different phytochemical metabolites especially at the aerial part of *Euphorbia* species like flavonoids, terpenoids, alkaloids, tannins, polyphenols and steroids. They have great role in different aspects of human demands at pharmacological and industrial activities. Most of them are used as drugs and commercial value products due to their active potential constituents **[16]**.

Phytochemical screening of *E. prolifera* and *E. spinidens* showed different terpenoids and flavonoids acquiring neuroprotective and immunomodulatory activities respectively **[17, 18]**. **Baniadam et al., (2014) [19]** studied the cytotoxicity of methane extract of *E. macrostegia* against tumor investigations like MCF-7 and MDA-MB48. Findings of *E. hirta* extracts indicated antimicrobial activities against different pathogenic Gram (+) and (-) strains **[20]** besides antioxidants properties that have defense mechanisms originating from different habitats **[21]**. Moreover *E. cornigera* and *E. fischeriana* expressed anthelmintic and antiviral activities respectively **[22]**. *E. milii* was candidate to fight different human diseases due to presence of tannins and flavonoids **[23]**.

The most robust and modern separated phytochemical technique is HPLC. It is able to separate

mixture of organic chemical compounds with high quality and measure the quantity precisely. It can analyze the natural products with standard purification and rapid processing. It provides phytochemical data to researchers who can describe the chemical properties and identify the classes of active fractions **[24]**.

According to the plenty of phytochemicals, Euphorbiaceae can live and adapt in many different zones; tropical, subtropical and temperate ones **[25]**.

Euphorbia spp. have unique morphological structures like pseudodanthial special type of inflorescence called cyathium, colorful tending and sub-tending bracts, cyathial nectar glands provided with petaloid appendages as well as succulent xerophytic stem protruding with horny thorns. Architecture leaf characterizations include many modified details on marginal configurations, venation types and leaf shape patterns. The distinct adapted morphology of the genus *Euphorbia* distinguishes its entire species and causes significant taxonomic segregation that needs more researches and studies. *Euphorbia* spp. are highly rich in secondary metabolites especially phytotoxins due to presence of milky or colored sap; the name of their family (spurge family) is emerged from it **[26]**.

In Saudi Arabia, there are nearly 40 species of the genus but in Jazan region that located at the southwestern part of Saudi Arabia, there are nearly 15 species **[27]** that most of them are succulent with sharp spines while others are neither succulent nor owing spines **[28]**.

The aim of this study is to evaluate the interrelations among four Euphorbia species at Jazan region by using two different categories; succulent with sharp spines; *E. ammak* (EA) and *E. fractiflexa* (EF) besides non-succulent without sharp spines; *E. granulata* (EG) and *E. hirta* (EH) upon modern taxonomic tool; chemotaxonomy besides the traditional one; morphology.

2. Materials and Methods

2.1. Collection of plant material and preparation

Four selected species of *Euphorbia*: *E. ammak, E. fractiflexa, E. granulata* and *E. hirta* were collected from mountains, high latitudes, valleys, and inhabited in-situ area respectively at Jazan region during autumn 2022. They were kept and identified through the herbarium investigator for College of Science, Jazan University, KSA 'JAZUH, Coordinates from the maps of Saudi Survey Authority' **(Table 1) (Figures 1 & 2)**.

The plant samples were analyzed to determine the morphological characters that were classified as qualitative *i.e.* the character presents or not as well as quantitative *i.e.* the character can be

measured. Micro-capillar ruler and magnified lens were used in analysis. After that, they were washed with dist. water 2-3 times then dried at 55^0C till constant weight finally cut into small pieces preluding to grind as powder sample for phytochemical analysis.

Table 1 Coordination and location of *Euphorbia* species collected.

species	Collecting Coordinates (Lat. N, long E.)	Locality
E. ammak	**17º20̓ N 43º08̓ E**	**Jabal Fayfa**
E. fractiflexa	**17º08̓ N 43º05̓ E**	**Jabal Abadil**
E. granulata	**17º11̓ N 43º10̓ E**	**Wadi Dahan,**
E. hirta	**17º25̓ N 42º35̓ E**	**Wadi Baysh**

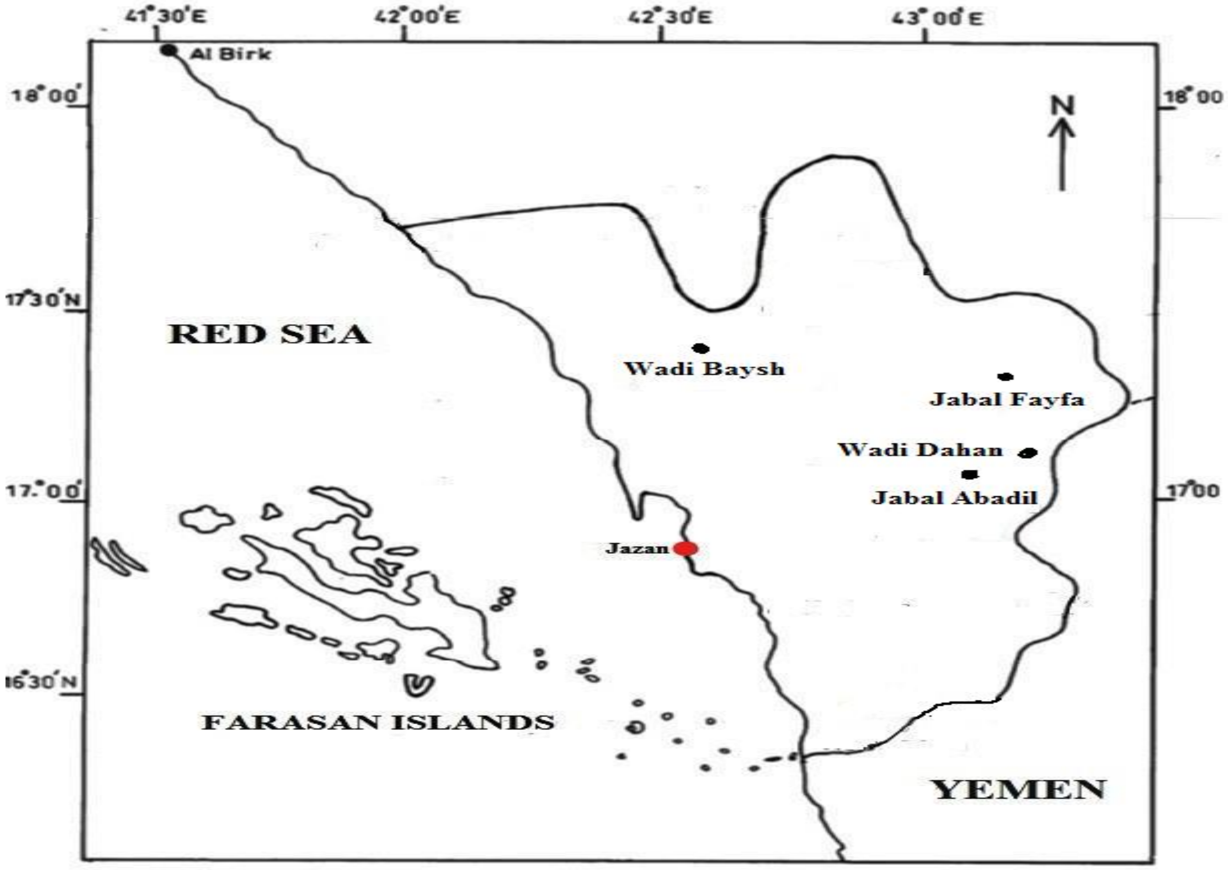

Fig. 1 Map of the studied area

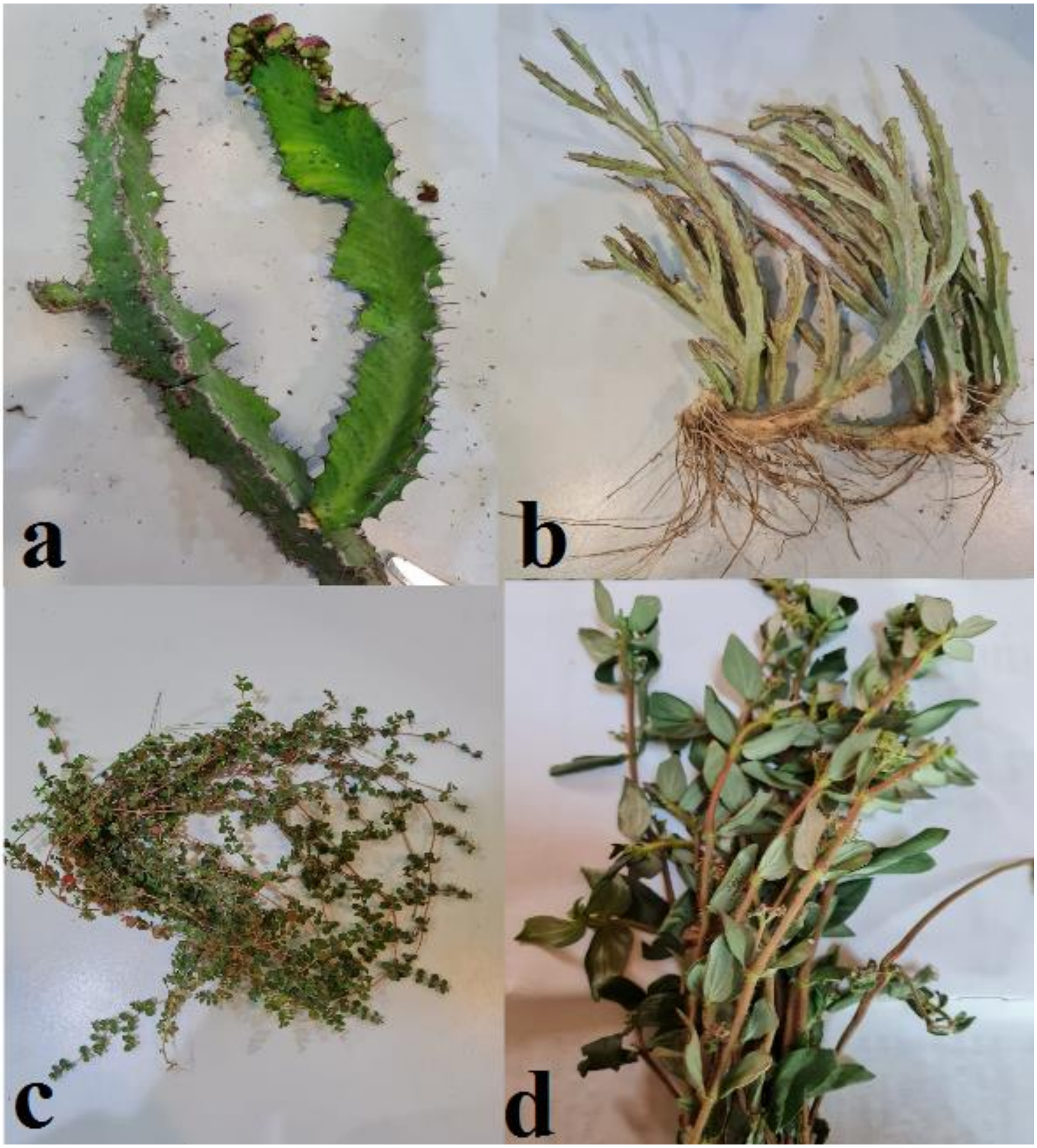

Fig. 2 The studied *Euphorbia* spp.; a. *E. ammak,* b. *E. fractiflexa,* c. *E. granulata*, d. *E. hirta.*

3. Estimation of phytochemical active constituents

Using the following techniques, the total active components of each of the following species; *E. ammak, E. fractiflexa, E. granulata,* and *E. hirta* were estimated separately.

3.1. Total phenolic content (TPC)

It was measured using the Folin-Ciocalteau technique **[29, 30]**. 3 ml of 10% solution was mixed with 5 µl of plant extract and 0.8 ml of 7.5% sodium bicarbonate. The reaction solution was incubated at room temperature at 30 min. The mixture's absorbance was determined at 765 nm using a Milton Roy (Spectronic 1201) spectrophotometer. A measure of the TPC was mg of gallic acid equivalents (GAE)/g of extract.

3.2. Total flavonoid content (TFC)

Chang *et al*. (2002) [3] technique was used to quantify TFC. In short, 3.9 ml of dist. water, 0.3 ml of 5% sodium nitrite solution, and 0.1 ml of extract were mixed. The mixture was left to react for 5 min before 0.3 ml of 10% aluminum chloride solution was added. After another 6 min of reaction, the mixture was treated with 2 ml of sodium hydroxide (1 mM-1). Finally, each sample received 2.4 milliliters of dist. water. Using a Milton Roy (Spectronic 1201) spectrophotometer, the absorbance was measured at 510 nm against a sample blank that had not undergone any reaction. The extract's TFC is given as mg of quercetine equivalents (QE) per gram of extract.

3.3. Content of total alkaloids

Using the methodology outlined by **Harbone (1973) [31]**, the total alkaloids of the aerial sections of *Euphorbia* plants were quantitatively quantified. 200 ml of 20% acetic acid in ethyl alcohol and 3 g of plant powder were mixed in a 250 ml conical flask. The conical was then covered and allowed to stand for 6 h using a water bath the plant extract was filtered and concentrated to 25% of its initial volume. To the concentrated extract, a concentrated NH_4OH solution was added drop by drop until full precipitation. After letting the whole mixture settle, the precipitate was gathered, filtered, and weighed.

3.4. Total saponin content

In a 250 ml conical flask, 25 ml of 20% ethanol was mixed with about 50 mg of the plant powder. In short, the suspension was continuously stirred while heated to 55 °C for 4 h. After the mixture was filtered, 50 ml of 20% ethanol were used to remove the residue once more. The mixture of extracts was concentrated to 10 ml at 90°C. In order to create a diethyl ether layer,

the concentrated extract was thoroughly agitated with 5 ml of diethyl ether in a separating funnel. The ether layer was then removed, allowing the aqueous layer to be recovered. There was another round of this cleansing. After adding 15 ml of n-butanol to the aqueous layer and shaking to create two layers, the butanol layer was collected for 3 times. 10 ml of 5% aqueous sodium chloride were used to completely wash the mixed n-butanol extracts twice. The saponin content was determined by evaporating the residual solution in a water bath, drying it in an oven, and weighing it **[24]**.

3.5. Total tannin content

It was calculated using the copper acetate method, which relies on quantitative tannin precipitation with copper acetate solution, copper tannate ignition to copper oxide, and residual copper oxide measurement. Two grams of plant powder were extracted by agitating two separate volumes of 100 ml each of acetone and water (1:1) for 1 h in a mechanical shaker, followed by filtering. After transferring the mixed extract into a 250 ml volumetric flask, dist. water was used to adjust the volume. After measuring out each extract and placing it in a 500 ml beaker, 30 ml of a 15% aqueous copper acetate solution was added while stirring. The ashless filter paper was used to collect the precipitated copper tannate, which was then fired in a porcelain crucible (the crucibles had been ignited to a constant weight at the same temperature beforehand). The residue was mixed with a few drops of nitric acid and re-ignited to a consistent weight. The following correlation was used to compute the weight of copper oxide and the proportion of tannin: For every 1g of CuO, there was approximately 1.305 g of tannins **[1]**.

4. HPLC analysis of phenolics and flavonoids

HPLC equipment (Agilent Series 1100; Agilent, USA) comprising an auto-sampling injector, solvent degasser, two LC pumps (series 1100), ChemStation software, and a UV/Vis detector (set at 250 nm for phenolic acids and 360 nm for flavonoids) was used to analyze phenolic and flavonoid compounds. C18 column (125 mm × 4.60 mm, 5 µm particle size) was used for the study. Using a gradient mobile phase of solvents A and B (A = methanol; B = acetic acid in water (1:25)], phenolic acids were isolated. For the first three minutes of the gradient procedure, the concentration was maintained at 100% B. This was followed by 50 % eluent A for the next 5 minutes after which concentration of A was increased to 80 % for the next 2 minutes and then reduced to 50 % again for the following 5 min detection wavelength at 250 nm. Flavonoids were separated by employing mobile phase of solvents C and D [C =

acetonitrile; D = 0.2 % (v/v) aqueous formic acid] with an isocratic elution (70: 30) program. The solvent flow rate was 1 ml/min, and separation was performed at 25°C. The injection volumes were 25 μl **[32, 33]**.

5. Scoring data with cluster analysis

In order to create a cluster analysis for *Euphorbia* spp., the morphological and phytochemical parameters were scored as binary matrix, with (0) denoting absence and (1) denoting presence. Distances were computed using a Gower coefficient modification, and a similarity matrix was created using Pclass. UPGMA was used for hierarchical nest clustering that was sequentially agglomerative **[34]**.

6. Statistical analysis

Duncan's range test was used to determine whether the results of quantitative phytochemical compounds were significant or not. It evaluated to differentiate among plant species in presence of a, b, c codes by conducting SPSS software (version 22) for Windows **[35]**.

7. Results and Discussion

7.1. Morphological analysis

Morphological characters included different aspects of plant species. They indicated the plant habit, life span and growth. Moreover, the reproductive plant parts were present in this investigation (fruit and inflorescence). Each plant part occupied different percent for presentation; stem; 31.82%, flowers; 27.27%, fruit; 22.73%, leaf; 9.09% and root; 4.54%. The characters were divided as qualitative like color, branching and others besides quantitative like fruit dimensions and pedicle length etc. The macro parameter; qualitative character possessed 81.82% while the micro parameter; quantitative character possessed 18.18%. The similarity parameter is considered the common character among studied plant species. It was 9.09% while the dissimilarity parameter was 90.91%. Unique parameter is the character which is only restricted to one plant species not present in the others. It was 27.27% distributed in only three species; *E. fractiflexa, E. granulata* and *E. hirta.* Furthermore, *E. granulata* occupied the highest value; 50% on the contrary, *E. hirta* had the lowest one; 16.67% **(Table 2)**.

Table 2 Morphological characters of studied plant species

	Morphological character	*E. ammak*	*E. fractiflexa*	*E. granulata*	*E. hirta*
1. Stems	**Branching**	Many	Many	Many	Sparingly
	**Surface**	Spineless	Spiny	Spineless	Spineless
	**Color**	Light green	Light green	Dark green	Dark green
	**Succulence**	Succulent	Succulent	Non-Succulent	Non-Succulent
	**Length**	Very tall	Tall	Very short	Short
	**Circumference**	Triangle	Triangle	Rounded	Rounded
	**Erection**	Erect	Decumbent	Decumbent	Erect
2. Inflorescences	**Color**	Yellow	Yellow	White	Yellow
	**Width**	7±0.05 mm	6±1.05 mm	1± 1.75 mm	< 1mm
	**Type**	Cyme	Cyme	Raceme	Cyme
	**Position**	Terminal	Terminal	Axillary	Axillary
	**Peduncle**	< 1 cm	< 1 cm	< 1 cm	> 1 cm
3. Fruits	**Color**	Reddish brown	Reddish brown	Gray	Gray
	**Type**	Capsule	Capsule	Capsule	Capsule
	**Dimensions**	8.5 x 13 mm	9 x 14 mm	1.5 x 1.5 mm	1.5 x 1.5 mm
	**Trichomes**	Glabrous	Glabrous	Pubescent	Pubescent
	**Shape**	Triangular, acute	Trigonous, rounded	Angular lobes	Angular lobes
	**Pedicel length**	2.5± 1.59 mm	3±1.78 mm	0.5±1.59 mm	1.2±1.48 mm
4. Roots	**Root length**	not deep	not deep	not deep	not deep
5. Leaves	**Lamina**	Leafless	Leafless	Compound	Simple
	**Leaf trichomes**	Absent	Absent	Present	Absent
6. Life span	**Duration**	Perennial	Perennial	Annual	Annual
7. Habit	**Life form**	Shrubs	Shrubs	herb	herb

7.2. Phytochemical analysis

The studied plant species exhibited different values with phytochemical data in all class of secondary metabolites. *E. granulata* was recognized in this investigation because it occupied the top in all classes. On one hand, it had the highest percent in phenolic compound, flavonoid, alkaloid, saponin, tannin content with 350.29 mg/g, 86.57 mg/g, 7.48%, 6.87% and 10.5% respectively. On the other hand, two plant species occupied the lowest level in different secondary metabolite classes.

E. hirta was the first one owing the lowest values in three classes; flavonoids (8.04 mg/g), alkaloids (3.46 %) and tannins (3.09%) while *E. fractiflexa* was the second one owing lowest values in two classes; phenolics (45.69 mg/g) and saponins (3.44%) **(Tables 3 & 4)**.

HPLC analyzes data for phenolic and flavonoid constituents of the four tested *Euphorbia* species were listed in table 3 and figures 3-5. Eleven phenolic (benzoic acid, catechol, caffeic acid, cinnamic acid, ferulic acid, gallic acid, pyrogallol, syringenic acid, *p*-coumaric acid, salicylic acid, and chlorogenic acid) and seven flavonoid (apigenin, kaempferol, luteolin, myricetin, naringin, quercetin, and rutin) compounds were identified from the four species with different relative percent.

Caffeic acid, gallic acid, and syringenic acid were the mutual phenolic acids among the four species, while apigenin and glycoside naringin were the mutual flavonoids.

EA showed a high percentage of caffeic acid, gallic acid, and apigenin, while EH showed the high percentage of syringenic acid and EF showed the high percentage of apigenin among the four species. Benzoic acid, cinnamic acid, salicylic, and kaempferol were the mutual compounds between EF, EG and EH species, while *p*-coumaric acid, luteolin, and quercetin were the mutual compound between EA, EG, and EH species.

Pyrogallol was the mutual phenolic between EA, EF, and EH, while ferulic acid was mutual between EA and EH only.

Catechol was the mutual phenolic between EF and EG. Chlorogenic acid, and Rutin were identified only from the EA species. Myricetin flavonoid was identified only from EF species.

EA had the highest values for all identified phytochemicals; *p*-Coumaric acid (21.56 µg/g) and Apigenin (21.76 µg/g) while the lowest value was obtained by EH at Gallic acid (0.98 µg/g).

The similarity parameter among identified phytochemicals was 44.44%. Negative unique parameter (NUP) is considered the parameter that is absent in a specific plant species but present in others. It occupied 33.33% and presented equally in two species: EA and EF.

Nevertheless, there was another type of parameter; positive unique parameter (PUP). It is considered that the parameter present only in one species not others. It was 11.11% and located only at EA in presence of Chlorogenic acid and Rutin **(Table 5) (Figures 3-6)**.

Table 3 Total phenolics and flavonoids contents

Sample Code	Total Phenolics mg/g					Total Flavonoids (mg/g)				
	1st rep.	2nd rep.	3rd rep.	Mean	S.D. (±)	1st rep.	2nd rep.	3rd rep.	Mean	S.D. (±)
E. ammak	136.56	130.41	125.92	130.96[ab]	5.34	83.20	78.35	76.56	79.37[a]	3.44
E. fractiflexa	39.65	51.29	46.13	45.69[c]	5.83	33.02	31.79	28.14	30.98[b]	2.54
E. granulata	371.25	314.69	364.93	350.29[a]	30.99	86.57	88.23	84.91	86.57[a]	1.66
E. hirta	73.84	71.39	80.27	75.17[c]	4.59	6.92	8.17	9.04	8.04[c]	1.07

The assay was performed in triplicate

Table 4 Total content of alkaloids, saponin, and tannins

	Total alkaloids content			Total Saponin contents			Total tannins contents			
Sample ID	SW (g)	PW (g)	%	SW (g)	PW (g)	%	SW (g)	PW (g)	TW (g)	%
E. ammak	2.504	0.1169	4.66[ab]	2.05	0.0784	3.80[ab]	2.00	0.09609	0.1254	6.27[ab]
E. fractiflexa	2.513	0.1656	6.59[a]	2.03	0.0699	3.44[ab]	2.03	0.1314	0.1715	8.57[ab]
E. granulata	2.523	0.1886	7.48[a]	2.07	0.1423	6.87[a]	2.07	0.16122	0.2104	10.5[a]
E. hirta	2.519	0.0872	3.46[ab]	2.06	0.1102	5.37[a]	2.06	0.05118	0.0668	3.09[c]

SW = weight of sample; PW = weight of precipitate; TW = weight of tannins

Table 5 Relative percent of phenol and flavonoid components of Euphorbia species methanolic extracts screened by HPLC

#	Compound Name	**Concentration µg/g**			
		E. ammak	*E. fractiflexa*	*E. granulata*	*E. hirta*
Phenolic compounds					
1	Benzoic acid	-	9.22^{a}	5.11^{ab}	3.85^{b}
2	Catechol	-	5.1	4.18	
3	Caffeic acid	9.36^{a}	7.22^{a}	$5.6a^{b}$	2.1^{b}
4	Cinnamic acid	-	1.19^{ab}	3.41^{a}	1.06^{ab}
5	Ferulic acid	5.14^{b}	-	-	15.44^{a}
6	Gallic acid	18.64^{a}	8.23^{b}	15.54^{a}	0.98^{c}
7	Pyrogallol	0.54^{c}	2.61^{c}	-	17.37^{a}
8	Syringenic acid	6.41^{a}	7.08^{a}	3.22^{c}	10.41^{a}
9	*p*-Coumaric acid	21.56^{a}	-	2.74^{c}	2.02^{c}
10	Salicylic acid	-	7.41^{a}	8.07^{a}	4.63^{ab}
11	Chlorogenic acid	5.07	-	-	-
Flavonoid compounds					
12	Apigenin	21.76^{a}	5.2^{c}	9.85^{b}	12.74^{b}
13	Kaempferol	-	8.19^{a}	4.24^{ab}	5.18^{ab}
14	Luteolin	10.41^{a}	-	6.32^{b}	15.26^{a}
15	Myricetin	-	6.78	-	-
16	Naringin	8.95^{ab}	15.36^{a}	8.57^{ab}	4.76^{b}
17	Quercetin	6.29^{a}	-	5.44^{a}	6.41^{a}
18	Rutin	6.41	-	-	-

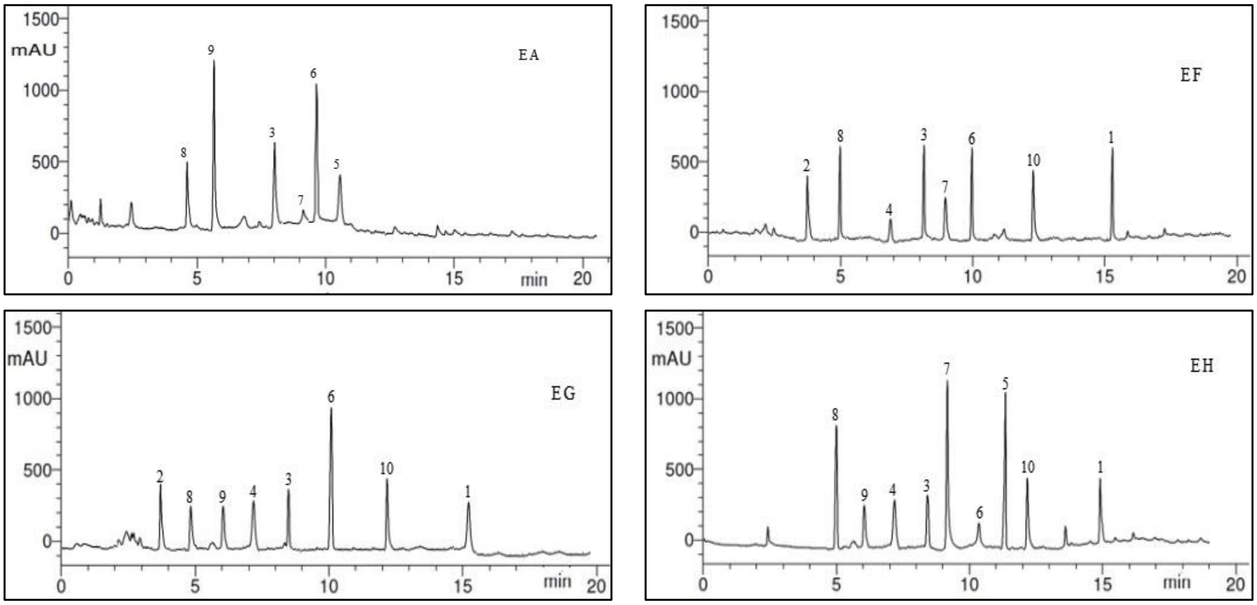

Fig. 3 HPLC Chromatogram of the phenolic components of *E. ammak* (EA), *E. fractiflexa* (EF), *E. granulata* (EG), and *E. hirta* (EH) methanolic extracts Peaks: 1, benzoic acid; 2, catechol; 3, caffeic acid; 4, cinnamic acid, 5, ferulic acid; 6, gallic acid; 7, pyrogallol; 8, syringenic acid; 9, *p*-coumaric acid; 10, salicylic acid.

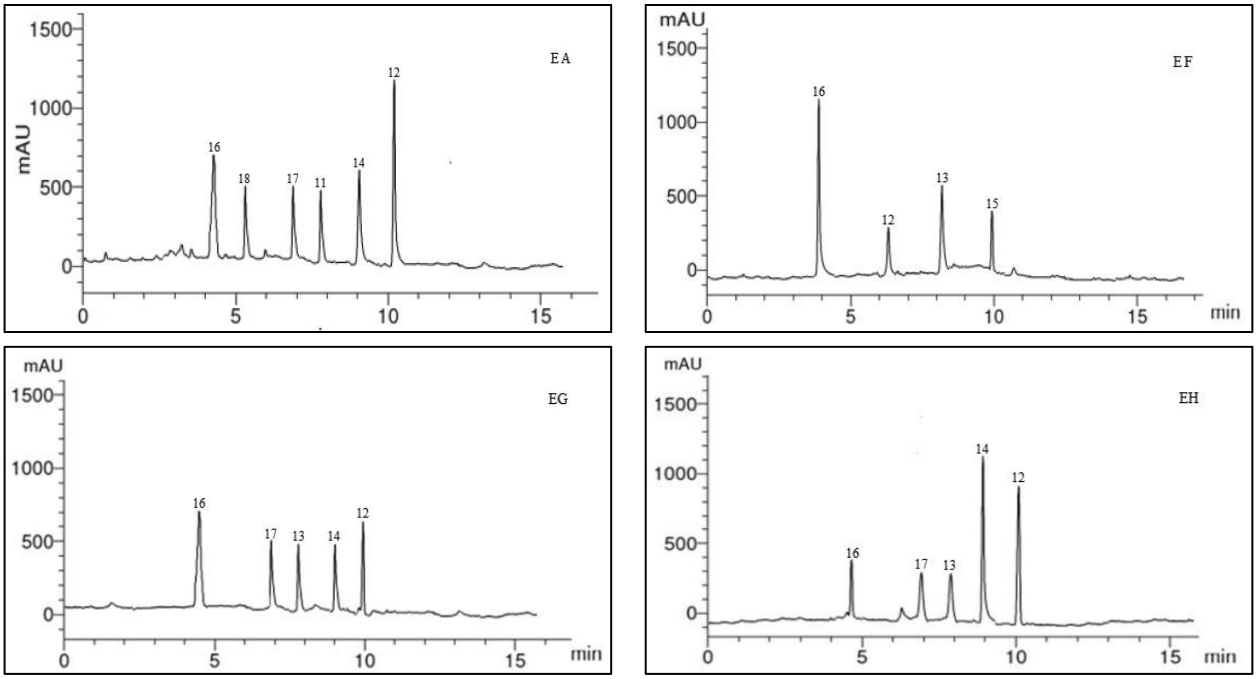

Fig. 4 HPLC Chromatogram of the flavonoid components of *E. ammak* (EA), *E. fractiflexa* (EF), *E. granulata* (EG), and *E. hirta* (EH) methanolic extracts. 11, chlorogenic acid; 12, apigenin; 13, kaempferol; 14, luteolin; 15, myricetin; 16, naringin; 17, quercetin; 18, rutin.

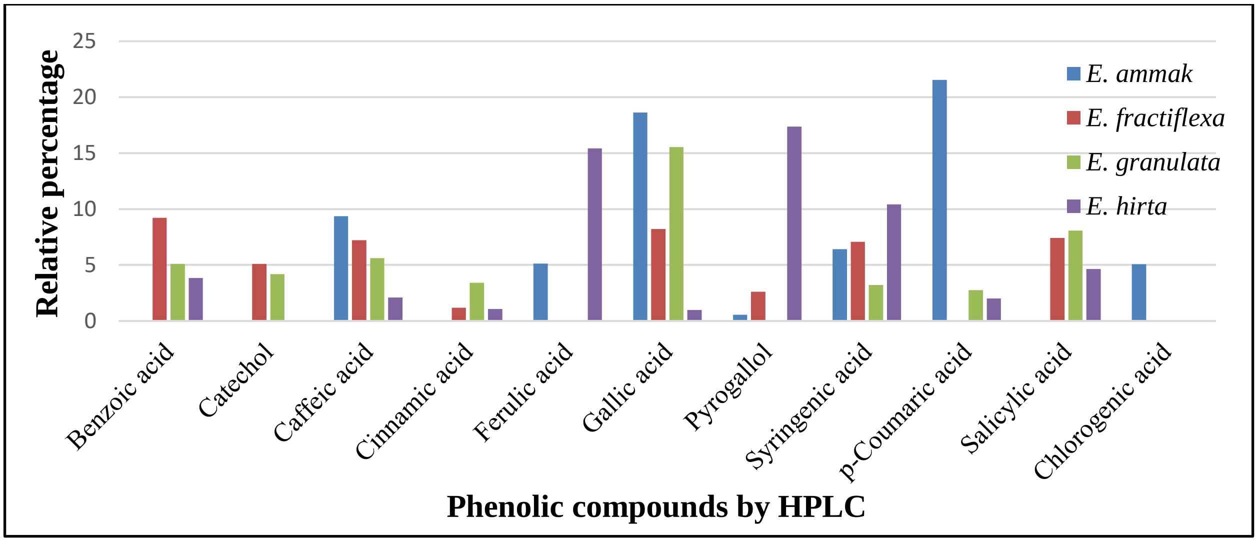

Fig. 5 Relative percent of phenolic compounds of *Euphorbia* spp. using HPLC.

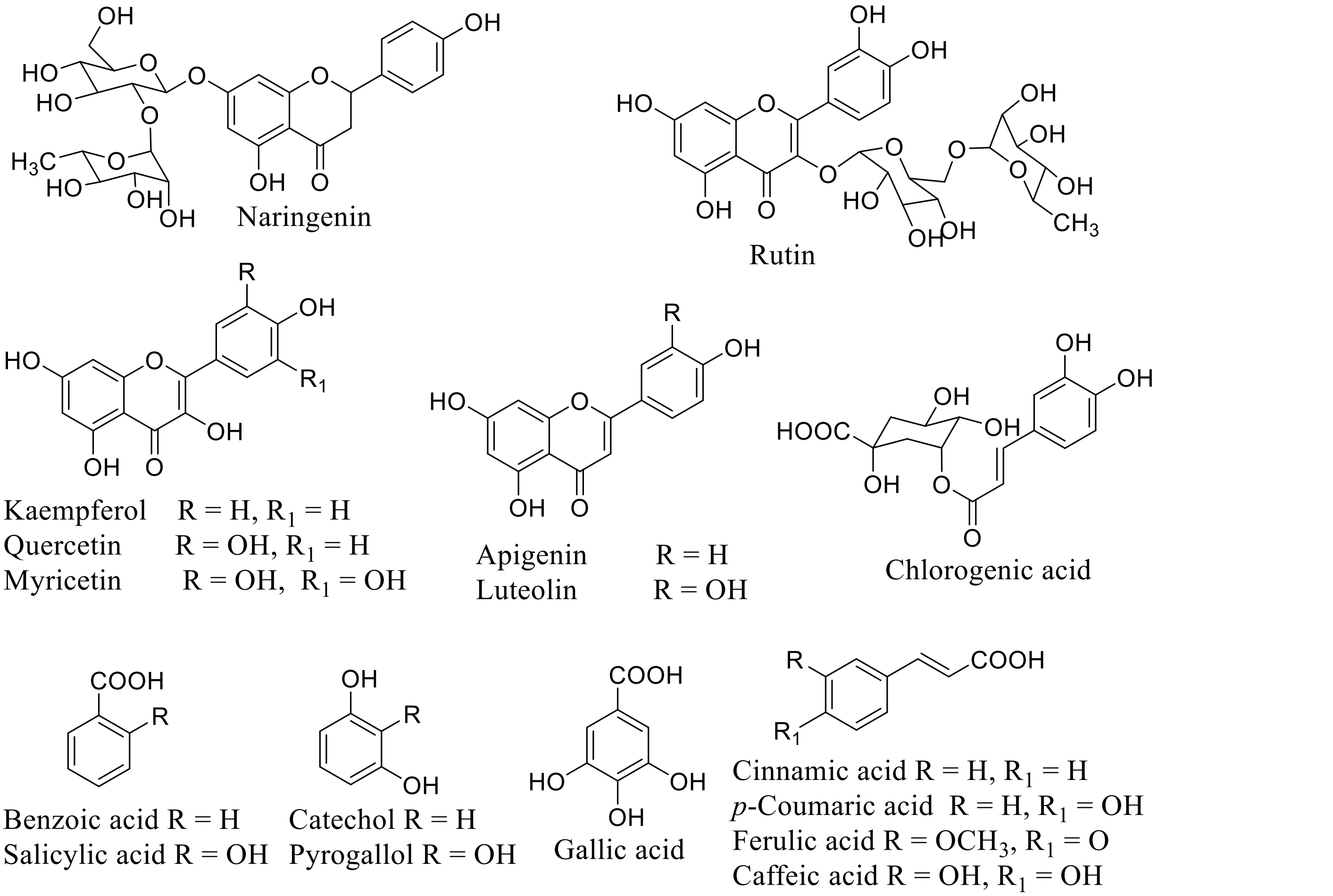

Fig. 6 The chemical structures of the identified phenolic and flavonoids compounds from EA, EF, EG, and EH methanolic extracts screened by HPLC.

* EA = *E. ammak*; EF = *E. fractiflexa*; EG = *E. granulata*; EH = *E. hirta*

7.3. Scoring analysis

Total 45 traits for both morphological and phytochemicals were scored as binary matrix to differentiate among studied plant species. The binary matrix denoted as (0) absent and (1) present. For morphological characters, the common character was represented with (1) while the unique or less common was (0). On the other hand, total active constituent content was represented with (1) donation for highest values while (0) donation was the lowest ones. Moreover, absent phytochemical compound from HPLC denoted with (0) meanwhile all present phytochemicals with different concentrations were denoted with (1).

The similarity matrix was obtained to indicate that the highest score was between *E. granulata* and *E. hirta* (0.69) however, the lowest one was between *E. ammak* and *E. granulata*.

The phenogram showed the interrelations among studied plant species. The taxonomic deviation for them was at 1.06 taxonomic unit where two groups appeared. The first group was at 1.90 included *E. ammak* and *E. fractiflexa* while the another group was at 1.52 included the rest of plant species **(Table 6) (Figure 7)**.

Table 6 Similarity matrix among *Euphorbia* spp.

	E. ammak	***E. fractiflexa***	***E. granulata***	***E. hirta***
E. ammak	**1.00**	**0.62**	**0.38**	**0.44**
E. fractiflexa	**0.62**	**1.00**	**0.44**	**0.42**
E. granulata	**0.38**	**0.44**	**1.00**	**0.69**
E. hirta	**0.44**	**0.42**	**0.69**	**1.00**

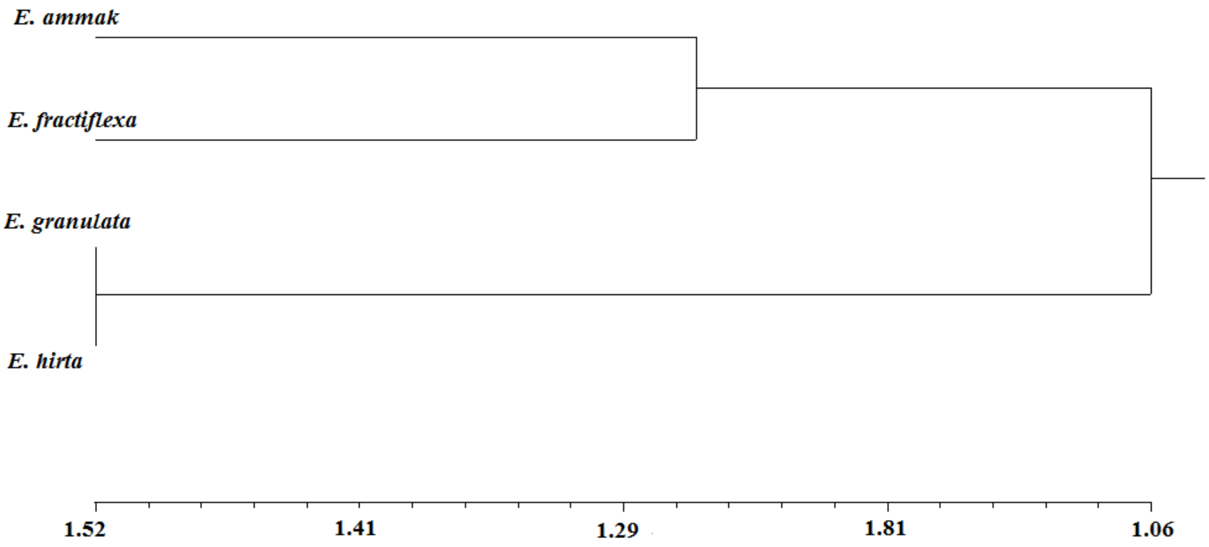

Fig. 7 Phenogram for the studied *Euphorbia* spp.

Taxonomy of the genus *Euphorbia* faces discerned challenges and implications owing to insufficient taxa collection, homoplasious and obscure morphological traits in addition to different biogeography. Other taxonomic tool like chemotaxonomy is integrated to enhance phylogenetic interrelationships and evolution among them **[36, 37]**.

Chemotaxonomy is regarded as the efficient and vital tool for plant taxonomy. Combining between modern and traditional classifications can promote the accurate taxonomic methodology **[38]**.

Although the studied plant species were divided into two groups obviously not based only on morphological characters but confirmed with phytochemicals, *E. granulata* was present as a transition species among studied ones. It had the majority of identified phytochemical contents though it had not any milky sap. It had the majority of identified phytochemicals by HPLC in addition to that *E. ammak* (succulent with milky sap) had also other separated phytochemicals not presented in *E. granulata* and vice versa. Although this distant variation in morphological characters between them *i.e. E. ammak* is tree, succulent with milky sap on the contrary, *E. granulata* is decumbent, ephemeral, non-succulent without milky sap, this situation confirmed that there is a link between them leading both to be in one family. This distant variation may refer to the nature of habitats. Adaptation of plant species to its habitat may protrude to be more variable than other plant species belonging to the same family. Phytochemicals reported to help the plant to resistant and withstand in its habitat **[39, 40]**.

This systematic investigation revolves around the importance of phytochemical secondary metabolites at plant taxonomy aspects as well as the link between these compounds and specific environmental locality with different responses.

World ecological distribution and various morphological variability of *Euphorbia* spp. induce different categories of secondary metabolites that attract most of researchers in taxonomical, pharmacological, medicinal and physiological studies since modern and ancient times.

Continuous phytochemical screening for different species belonging to the same genus at different localities all over the world encourages collecting data serving humanity at different fields of life.

Presence of phytochemicals with high concentrations in the form of secondary metabolites encourages the researchers to use this family in pharmacological and medicinal studies. Even the same plant habitat and behavior exposed different concentrations and classes of secondary metabolites that providing more varied phytochemical classes if located in different or harsh environments.

8. Conclusion

The study of phytochemicals should be done under taking into consideration the habitat of plant species especially if the plant species belonging to remarkable family with rich secondary metabolites. Not all milky sap is rich in phytochemicals but it may contain other more toxic compounds in plenty quantity. Chemotaxonomy should be undergone side by side with traditional taxonomy to give the full picture of interrelations among plant species. The current characterization of the secondary metabolites is a beneficial tool for discrimination between the four closely related *Euphorbia* species that supports their morphological variation.

Declaration of Competing Interest

The authors declare that they have no known competing financial interests or personal relationships that could have appeared to influence the work reported in this paper.

Availability of data

All datasets were achieved, used, and analyzed in the current study. They are included in this manuscript.

References

[1] Ali A. A., Ross S. A., Mesbah M. K., El Moghazy S. A. Phytochemical study of *Limonium axillare* (Forssk.), Bull. Fac. Pharm., 29 (3) (1991) 59-62.

[2] Alfarhan A. H., Al-Turki T. A., Basahy, A. Y. Flora of Jizan region, Final Report Supported by King Abdulaziz City for Science and Technology 1(2) (2005) 545.

[3] Chang C.-C., Yang M.H.,Wen H.M., Chern J.C. Estimation of total flavonoid content in propolis by two complementary colorimetric methods. J. Food Drug Anal. 10 (2002) 178–182.

[4] Fatope M.O., Zeng L., Ohayaga J.E., Shi G. McLaughlin, J.L. Selectively cytotoxic diterpenes from *Euphorbia poisonii*, J. Med. Chem. 39 (1996) 1005–1008.

[5] Murugan S., Anand R., Uma-Devi P., Vidhya N., Rajesh K.A. Efficacy of *Euphorbia milli* and *Euphorbia pulcherrima* on aflatoxin producing fungi (*Aspergillus flavus* and *Aspergillus parasiticus*), Afr. J. Biotechnol. 6 (2007) 718–719.

[6] Ma Q.G., Liu W.Z., Wu X.Y., Zhou T.X., Qin G.W. Chemical studies of Lang-Du, a traditional Chinese medicine. 1. Diterpenoids from *Euphorbia fischeriana*, Phytochemistry 44 (1997) 663–666.

[7] Hezareh M. Prostratin as a new therapeutic agent targeting HIV viral reservoirs, Drug News Perspect. 18 (2005) 496–500.

[8] Ernst M., Grace O. M., Saslis-Lagoudakis C. H., Nilsson N., Simonsen H. T., Rønsted N. Global medicinal uses of *Euphorbia* L. (Euphorbiaceae), J. Ethnopharmacol. 176 (2015) 90–101.

[9] Amtaghri S., Akdad M., Slaoui M., Eddouks M. Traditional Uses, Pharmacological, and Phytochemical Studies of *Euphorbia*: A Review, Curr. Top. in Med. Chem. 22(19) (2022) 1553–1570.

[10] Qing-Wen S., Xiao-Hui S., Hiromasa K. Chemical and pharmacological research of the plants in genus *Euphorbia, Chem Rev.* 108 (2008) 4295–4327.

[11] Vasas A. & Hohmann J. *Euphorbia* Diterpenes: Isolation, Structure, Biological Activity and Synthesis, Chem. Rev. 114 (17) (2014) 8579–8612.

[12] Salehi B., Iriti M., Vitalini S., Antolak H., Pawlikowska E., Kręgiel D., Sharifi-Rad J., Oyeleye S.I., Ademiluyi A.O., Czopek K. et al. *Euphorbia*-derived natural products with potential for use in health maintenance, Biomolecules 9 (2019) 337.

[13] Sofrenić I., Anđelković B., Gođevac D., Ivanović S., Simić K., Ljujić J., Tešević V., Milosavljević S. Metabolomics as a Potential Chemotaxonomical Tool: Application on the Selected *Euphorbia* Species Growing Wild in Serbia, Plants 12 (2023) 262.

[14] Kemboi D., Peter X., Langat M., Tembu J. A Review of the Ethnomedicinal Uses, Biological Activities, and Triterpenoids of *Euphorbia* Species, Molecules 25 (17) (2020) 4019.

[15] Jasbi A.R. Chemistry and biological activity of secondary metabolites in Euphorbia from Iran, Phytochemistry 67 (2006) 1977–1984.

[16] Waseem Ahmad, Shilpa Singh and Sanjay K. (2017). Phytochemical Screening and antimicrobial study of *Euphorbia hirta* extracts, J. Med. Plants Stud. 5(2) (2017) 183-186.

[17] Xu J, Jin D, Guo P, Xie C, Fang L, Guo Y. (2012). Three new myrsinol diterpenes from *Euphorbia prolifera* and their neuroprotective activities, Molecules 17 (2012) 9520-9528.

[18] Ghannadian M, Akhavan A, Abdalla OM, Ayatollahi AM, Mohammadi-kamalabadi M, Ghazanfari H. Triterpenes from *Euphorbia spinidens* with immunomodulatory activity, J. Pharm. Res. Sci. 8(3) (2013) 205-210.

[19] Baniadam S, Rahiminejad MR, Ghannadian M, Saeidi H, Ayatollahi AM, Aghaei M. (2014). Cycloartane Triterpenoids from *Euphorbia Macrostegia* with their Cytotoxicity against MDA-MB48 and MCF-7 Cancer Cell Lines, *Iranian* J. Pharm. Res. 13 (1) (2014)135-141.

[20] Sudhakar M, Rao CV, Rao PM, Raju DB, Venkateswarlu Y. Antimicrobial activity of *Caesalpinia pulcherrima*, *Euphorbia hirta* and *Asystasia gangeticum*, Fitoterapia 77(5) (2006) 378-380.

[21] Lavanya R., Monisha M., Narmatha M., Fouzeeya begum S. R., Sathiya Savithri J. Phytochemical screening of *Euphorbia hirta* L. and antioxidant properties, Int. J. Bot. Stud. 7 (3) (2022) 228-234.

[22] Olounladé Abiodoun Pascal, Azando Erick Virgyle Bertrand, Tchétan Esaïe, Hounzangbé-Adoté Mawulé Sylvie and Attakpa Yatchégnon E. A review of the ethnomedical uses, phytochemistry and pharmacology of the *Euphorbia* genus, The Pharma. Innovation J. 6(1) (2017) 34-39.

[23] Muhammad Q., Syed N. G., Shahid F., Abdur R., Rifat N. S., Samreen P. Preliminary Comparative Phytochemical Screening of *Euphorbia* Species, American-Eurasian J. Agric. Environ. Sci. 12 (8) (2012) 1056-1060.

[24] Ajiboye BO., Ibukun EO., Edobor G., Ojo AO., Onikanni SA. Qualitative and quantitative analysis of phytochemicals in *Senecio biafrae* leaf, Inter. J. Invent. Pharm. Sci. 2013;1(5) (2013) 428-32.

[25] Abdel Aziz A. Fayed, Mohamed S. Ahamed, Ahamed M. Faried, Mona H. M. Leaf Morphology and Venation Patterns of *Euphorbia* L. (Euphorbiaceae) in Egypt with Special Notes on Their Taxonomic Implications, Jordan J. Biol. Sci. 13 (2) (2020) 165-176.

[26] Lee S. R., Oh A., Son D. C. (2024). Characterization, comparison, and phylogenetic analyses of chloroplast genomes of *Euphorbia* species. Sci. Rep. 14 (1) (2024) 15352.

[27] Mihail A., Viktorija M., Liljana K.G., Liljana G.T. Review of the anticancer and cytotoxic activity of some species from the genus *Euphorbia*, Agric. Conspec. Sci. 84 (2019) 11–23.

[28] Xu Z.H., Sun J., Xu R.S., Qin G.W. Casbane diterpenoids from *Euphorbia bracteolate*, Phytochemistry 49 (1998) 149–151.

[29] Singleton V. & Rossi J.A. Colorimetry of total phenolics with phosphomolybdicphosphotungstic acid reagents, Am. J. Enol. Vitic. 16 (1965) 144–158.

[30] Singleton VL., Orthofer R., Lamuela-Raventos RM. Analysis of total phenols and other oxidation substrates and antioxidants by means of Folin–Ciocalteau reagent, Meth. Enzymol. 299 (1999) 152-78.

[31] Harbone J. B. Phytochemical methods, a guide to modern techniques of plant analysis. Chapman and Hall, Ltd. London. (1973) 185-186.

[32] Lin Y. L., Chen Y. L., Liang Y. C., Lin J. K. Composition of Polyphenols in fresh Tea Leaves and Associations of their Oxygen Radical Absorbing Capacity with Antiproliferative Actions in Fibroblast Cells, J. Agri. Food Chem. 44 (1996) 1387-1394.

[33] Kuntic V., Pejic N., Ivkovic B. Isocratic RP-HPLC method for rutin determination in solid oral dosage forms, J. Pharm. Biomed. Anal. 43(2) (2007) 718–72.

[34] Abdullah Mashraqi, Mohamed A. Al Abboud, Khatib Sayeed Ismail, Yosra Modafer, Mukul Sharma, A. El-Shabasy Comparative phytochemical study of Artemisia sp. in the Middle East: A focus on antimicrobial activities and GC-MS analysis in *A. absinthium* L. Jazan, KSA and *A. herba-alba* Asso Sinai, EGY, J. Med. plants Byprod. 3 (2024) 479-503.

[35] Deya El-deen M.Radwan and Ahmed E. El-Shabasy Comparative Analysis of Five *Heliotropium* species in Phenotypic Correlations, Biochemical Constituents and Antioxidant Properties, Catrina 21(1) (2020) 1-8.

[36] Brian L. Dorsey, Thomas Haevermans, Xavier Aubriot, Jeffery J. Morawetz, Ricarda Riina, Victor W. Steinmann, Paul E. B. Phylogenetics, morphological evolution, and classification of *Euphorbia* subgenus *Euphorbia*, Taxon 62 (2) (2013) 291–315.

[37] Dalia G. Gabr, AlQahtani R. A Review on: Morphological, Phytochemical and Medical Important of Some Wild Euphorbiaceae Poisonous Plants, Haya. Saudi J. Life Sci. 7(11) (2022) 299-313.

[38] Atta E., T. Al faifi, A. El-Shabasy Chemotaxonomic and morphological classification of six *Indigofera* species in Jazan region, KSA, Journal of Saudi Chemical Society 26 (3) (2022) 101476.

[39] Sharovikj Ivanova A., Schönswetter P., Kostadinovski M., Barfuss M. H. J., Ćušterevska R., Frajman B. Integrative taxonomy reveals cryptic diversity within the *Euphorbia nicaeensis alliance* (Euphorbiaceae) in the central Balkan Peninsula, Front. Plant Sci. 16 (2025) 1558466.

[40] Jassim A. H. & Al-Newani H. R. H. Chemotaxonomy Significant in Delimitation of Six Taxa of *Euphorbia* L. in Iraq. IOP Conference Series: Earth and Environmental Science 1262(5) (2023) 052019.
