# Supplementary material for: Morphological and chemotaxonomical characterization of some species of the genus Euphorbia L. in Jazan region, KSA
Source: PLoS One. 2025 Nov 5;20(11):e0335519. doi: 10.1371/journal.pone.0335519 (PMC12588485; doi:10.1371/journal.pone.0335519)
Supplement: Attachment — Submitted filename: final report.docx [file pone.0335519.s002.docx]

Reviewer Report

Journal: POLS Journal

Manuscript Title: Chemotaxonomic and Morphological Delimitation of Selected Euphorbia Species from Jazan Region, Saudi Arabia

Reviewer: Dr. Amir Shahbaz

Date: June 25, 2025

**General Assessment**

The manuscript presents an integrative study on the classification of four Euphorbia species using both traditional morphological and modern chemotaxonomic approaches. The authors employed quantitative phytochemical analyses (including HPLC profiling of phenolics and flavonoids) and morphological scoring to assess interspecific relationships. The research topic is timely and relevant, particularly for floristic, taxonomic, and phytochemical studies within arid regions.

# **Major Revisions Needed**

1. Language and Grammar: The manuscript needs thorough English language editing by a native or fluent scientific editor.
2. . Scientific Terminology: Replace vague or incorrect terms with standardized scientific terminology.
3. Data Presentation: Improve table formatting and figure resolution; ensure clarity in HPLC chromatograms.
4. Introduction and Background: Improve paragraph transitions and clearly state the rationale for species selection.
5. Conclusion: Revise to avoid generalizations and ensure scientific clarity.

# Minor Points

- Ensure species names are italicized throughout.
- Correct and standardize reference formatting.
- Improve phenogram resolution and labeling.
- Clarify methodology in scoring matrix.

# Recommendation

Major Revision Required - The manuscript offers valuable taxonomic insights but needs substantial revision in language, terminology, and presentation before it can be considered for publication.
