# Supplementary material for: Morphological and chemotaxonomical characterization of some species of the genus Euphorbia L. in Jazan region, KSA
Source: PLoS One. 2025 Nov 5;20(11):e0335519. doi: 10.1371/journal.pone.0335519 (PMC12588485; doi:10.1371/journal.pone.0335519)
Supplement: Attachment — Submitted filename: Response to Reviewers.docx [file pone.0335519.s004.docx]

**Response to Reviewers**

PONE-D-25-25839

Morphological and chemotaxonomical characterization of some species of the genus Euphorbia L. in Jazan region, KSA

**Authors followed all editor and reviewers’ comments and answer each comment with red color**

**Editor’s comments**

1. Please ensure that your manuscript meets PLOS ONE's style requirements, including those for file naming.

I follow PLOS ONE’s style requirements

2. In your Methods section, please provide additional information regarding the permits you obtained for the work. Please ensure you have included the full name of the authority that approved the field site access and, if no permits were required, a brief statement explaining why.

These plant species presented at valleys and mountains along highways besides inside the city along street pavements as natural wild plants

3. Please ensure that you refer to Figure 3, 4, 5, 6 in your text as, if accepted, production will need this reference to link the reader to the figure.

OK

4. We require you to present written permission from the copyright holder to publish Figure (1) specifically under the CC BY 4.0 license, or (2) remove the figures from your submission

Figure (1) is from my design that was used at many published papers belonging to me as corresponding author. Here below the links of these published papers:

<https://www.sciencedirect.com/science/article/pii/S1319610322000588?fbclid=IwQ0xDSwMMySlleHRuA2FlbQIxMAABHjX-Aq8GYskEtRhEfGWbHC9vJ_z_W2Jl3EQfCQmn4Hx3lbszU_OR4b4C4mFo_aem_bFEVXJSUe4Y-O5S7gUhvIA&sfnsn=wa>

<https://scholar.google.com/citations?view_op=view_citation&hl=ar&user=08SOHxgAAAAJ&citation_for_view=08SOHxgAAAAJ:Tyk-4Ss8FVUC&fbclid=IwQ0xDSwMMyfJleHRuA2FlbQIxMAABHsVyeuYMcjtFoVbfEhcrqld51i8Nwp4QTc2-AoBN_xnrKui1aMeMpPPGc0dH_aem_Og1ewmhERaDM8TH9CcMLQg&sfnsn=wa>

<https://scholar.google.com/citations?view_op=view_citation&hl=ar&user=08SOHxgAAAAJ&citation_for_view=08SOHxgAAAAJ:Tyk-4Ss8FVUC&fbclid=IwQ0xDSwMMyfJleHRuA2FlbQIxMAABHsVyeuYMcjtFoVbfEhcrqld51i8Nwp4QTc2-AoBN_xnrKui1aMeMpPPGc0dH_aem_Og1ewmhERaDM8TH9CcMLQg&sfnsn=wa>

<https://scholar.google.com/citations?view_op=view_citation&hl=ar&user=08SOHxgAAAAJ&cstart=20&pagesize=80&citation_for_view=08SOHxgAAAAJ:ufrVoPGSRksC&fbclid=IwQ0xDSwMMyyxleHRuA2FlbQIxMAABHtGkfrEltFJkOu92y3riwHl3b_yOjpe7rLYlbKZ44e-STRDUel_3kS4UhLdy_aem_c7mQTboMlY1Z_sIaMzlp0Q&sfnsn=wa>

**Reviewers' comments:**

**Reviewer #1:** The data support the conclusion, the statistical analysis has been performed appropriately and rigorously, the authors made all data underlying the findings in their manuscript fully available, the manuscript presented in an intelligible fashion and written in Standard English.

The abstract must be include more results

It is modified according to reviewer#1

The materials should be abbreviation in abstract

It is modified according to reviewer#1

You must write full scientific name and in rest text you can abbreviation the genus name

It is modified according to reviewer#1

In results don't write the scientific name in wrong methods, all comments were written in the text.

It is adjusted according to reviewer#1

**Reviewer #2 Report**

# **Major Revisions Needed**

1. Language and Grammar: The manuscript needs thorough English language editing by a native or fluent scientific editor.

It is adjusted according to reviewer#2 with attached official report

2. . Scientific Terminology: Replace vague or incorrect terms with standardized scientific terminology.

It is adjusted according to reviewer#2

3. Data Presentation: Improve table formatting and figure resolution; ensure clarity in HPLC chromatograms.

It is adjusted according to reviewer#2

4. Introduction and Background: Improve paragraph transitions and clearly state the rationale for species selection.

It is adjusted according to reviewer#2

5. Conclusion: Revise to avoid generalizations and ensure scientific clarity.

It is adjusted according to reviewer#2

# Minor Points

- Ensure species names are italicized throughout.
- Correct and standardize reference formatting.
- Improve phenogram resolution and labeling.
- Clarify methodology in scoring matrix.

All minor points are done according to reviewer#2

# Corrections on text at attached pdf file

Each correction on text at attached pdf file is done with referring to detailed comment
